# Small-Molecule Compound SYG-180-2-2 to Effectively Prevent the Biofilm Formation of Methicillin-Resistant *Staphylococcus aureus*

**DOI:** 10.3389/fmicb.2021.770657

**Published:** 2022-01-07

**Authors:** Lulin Rao, Yaoguang Sheng, Jiao Zhang, Yanlei Xu, Jingyi Yu, Bingjie Wang, Huilin Zhao, Xinyi Wang, Yinjuan Guo, Xiaocui Wu, Zengqiang Song, Fangyou Yu, Lingling Zhan

**Affiliations:** ^1^Department of Laboratory Medicine, The First Affiliated Hospital of Wenzhou Medical University, Wenzhou, China; ^2^School of Pharmaceutical Sciences, Wenzhou Medical University, Wenzhou, China; ^3^Jiangxi Provincial Key Laboratory of Preventive Medicine, School of Public Health, Nanchang University, Nanchang, China; ^4^Department of Clinical Laboratory, School of Medicine, Shanghai Pulmonary Hospital, Tongji University, Shanghai, China

**Keywords:** MRSA, SYG-180-2-2, biofilm, cell adhesion, *icaA*

## Abstract

The resistance of methicillin-resistant *Staphylococcus aureus* (MRSA) has augmented due to the abuse of antibiotics, bringing about difficulties in the treatment of infection especially with the formation of biofilm. Thus, it is essential to develop antimicrobials. Here we synthesized a novel small-molecule compound, which we termed SYG-180-2-2 (C_21_H_16_N_2_OSe), that had antibiofilm activity. The aim of this study was to demonstrate the antibiofilm effect of SYG-180-2-2 against clinical MRSA isolates at a subinhibitory concentration (4 μg/ml). In this study, it was showed that significant suppression in biofilm formation occurred with SYG-180-2-2 treatment, the inhibition ranged between 65.0 and 85.2%. Subsequently, confocal laser scanning microscopy and a bacterial biofilm metabolism activity assay further demonstrated that SYG-180-2-2 could suppress biofilm. Additionally, SYG-180-2-2 reduced bacterial adhesion and polysaccharide intercellular adhesin (PIA) production. It was found that the expression of *icaA* and other biofilm-related genes were downregulated as evaluated by RT-qPCR. At the same time, *icaR* and *codY* were upregulated when biofilms were treated with SYG-180-2-2. Based on the above results, we speculate that SYG-180-2-2 inhibits the formation of biofilm by affecting cell adhesion and the expression of genes related to PIA production. Above all, SYG-180-2-2 had no toxic effects on human normal alveolar epithelial cells BEAS-2B. Collectively, the small-molecule compound SYG-180-2-2 is a safe and effective antibacterial agent for inhibiting MRSA biofilm.

## Introduction

*Staphylococcus aureus* is a pathogen that causes a variety of infections ranging from relatively benign to life-threatening infections including pneumonia, endocarditis, osteomyelitis, and sepsis (Cassat et al., 2007). Due to the use of antibiotics, drug-resistant strains have increased rapidly, especially methicillin-resistant *Staphylococcus aureus* (MRSA) which is difficult to treat and has a high mortality rate (Mole, 2013). It was reported that some MRSA had even developed resistance to vancomycin which is the most effective antibiotic for the treatment of MRSA (Cong et al., 2020). With the introduction of biomaterials such as artificial catheters and artificial joints, implant material-related infections frequently develop (Biedlingmaier et al., 1998), and they are usually persistent and multidrug-resistant, which brings a heavy burden to patients (Aslan and Yapar, 2015). The main cause of such infections is biofilm formation (Uruen et al., 2020). Biofilm is a kind of special colony structure formed by the encapsulation of a microorganism in its own secreted polymer (Hoiby et al., 2010), which will tend to resist both host clearance mechanisms and antibiotic therapy (Reed et al., 1986). Treatment with traditional antibiotics is ineffective to cope with the current severe drug resistance situation (Basnyat et al., 2015). Therefore, it is urgent to develop new drugs that cannot only effectively inhibit biofilm formation but also prevent bacterial mutations from developing drug resistance.

The formation of biofilm is a dynamic process, including initial adhesion, proliferation, maturation, and diffusion (Dufour et al., 2010). Initial adhesion is the first stage of biofilm formation. The most prominent cell wall-anchored proteins are microbial surface components that recognize adhesive matrix molecules (MSCRAMMs) that promote the binding of *S. aureus* to the host surface (Achek et al., 2020). MSCRAMMs including but not limited to fibronectin binding protein B (*fnbB*), laminin binding protein (*eno*), fibrinogen binding protein (*fib*), and encoding elastin binding protein (*ebpS*) promote the binding of *S. aureus* to the host surface (Nemati et al., 2009). The next stage of biofilm formation is the production of the extracellular matrix and cell proliferation. The extracellular polymeric substance (EPS) is mainly composed of polysaccharides, proteins, and extracellular DNA (eDNA) to protect cells (Lopez et al., 2010). A main component of the EPS is polysaccharide intercellular adhesin (PIA), which is mediated by the intercellular adhesin (*ica*) locus in *S. epidermidis* and *S. aureus* (Rohde et al., 2001; Fluckiger et al., 2005). In *S. aureus* ATCC 35556, mutation of the *ica* operon attenuated the production of PIA and lost the ability to form a strong biofilm (Cramton et al., 1999). *IcaR* is a negative regulator of the *ica* operon; inactivation of *icaR* augmented the transcription of the *icaAD* (Jefferson et al., 2004). In addition to *icaADBC*, *codY* also had an impact on PIA-dependent biofilm formation (Mlynek et al., 2020). The mutation of *codY* in *S. aureus* led to lower PIA production and less biofilm formation (Tu Quoc et al., 2007). The *S. aureus* Sae two component system involves the SaeS sensor histidine kinase and the SaeR response regulator, the former regulates the expression of exoproteins such as FnbA and FnbB, the latter is essential for the maturation of biofilms (Liu et al., 2016; Schilcher and Horswill, 2020). Besides, the accessory gene regulator (Agr) quorum sensing (QS) system is the most researched on the regulation system of staphylococcal biofilm formation (Yarwood et al., 2004). Part of QS-regulated genes are directly regulated by AgrA, for example, *psm*α and *psm*β (Jenul and Horswill, 2019). Phenol soluble modulins (PSM) expression can lead to the spread of biofilms, which in turn results in the systemic spread of biofilm infections (Peschel and Otto, 2013).

SYG-180-2-2 is a small molecule which consists of an indole ring, a selenyl group, and an amido group. Indoles have been identified as a privileged scaffold for the design of medicinal drugs (O’Connor and Maresh, 2006; Liu et al., 2009; Biersack and Schobert, 2012), 3-selenylindoles are a significant class of indole compounds as they are bioactive (Nogueira et al., 2004), and the amido group is a very important substituent in medicinal chemistry. Amide-containing compounds are widely present in natural products and pharmaceuticals, displaying a wide range of biological activities, such as anticancer and antiviral properties (Fatahala et al., 2017). Considering the dominance of 3-selenylindole and the amido group in nature and their impact in medicinal chemistry, we designed a novel compound SYG-180-2-2 containing these two frameworks. With our continuing interest in the discovery of new antibacterial agents (Yu et al., 2021), we have great interest in the exploration of the anti-bacterial activity of this novel compound. SYG-180-7 is similar to SYG-180-2-2 in structure and has strong hydrophobicity.

The purpose of this study was to investigate the effect of SYG-180-2-2 at a subinhibitory concentration (4 μg/ml) on the formation of MRSA biofilms and antibacterial mechanisms in order to evaluate the clinical potential of SYG-180-2-2 in the prevention and treatment of MRSA chronic infection.

## Materials and Methods

### The Synthesis of SYG-180-2-2 and SYG-180-7

**FIGURE 1:**
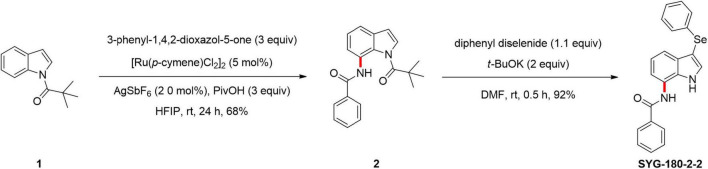
Synthetic process of SYG-180-2-2. 1: *N*-pivaloyl indoles. 2: *N*-(1-pivaloyl-1*H*-indol-7-yl) benzamide. SYG-180-2-2: desired product.

A mixture of *N*-pivaloyl indoles 1 (0.2 mmol), 3-phenyl-1,4,2-dioxazol-5-one (0.6 mmol), [RuCl_2_(*p*-cymene)]_2_ (5 mol%), AgSbF_6_ (20 mol%), PivOH (3 equiv), and HFIP (2 ml) was added in a 5 mL glass tube, which was stirred at room temperature for 24 h (Sheng et al., 2021). The reaction was stopped, and it was mixed with water and dichloromethane. The reaction mixture was extracted three times with dichloromethane. The combined organic layer was washed twice with a small amount of water, dried over anhydrous magnesium sulfate, and filtered. The filtrate was evaporated under a vacuum, and the residue was purified by flash column chromatography on silica gel (eluting with petroleum ether-ethyl acetate) to provide the desired product 2. A mixture of 7-amido indoles 2 (0.2 mmol), diphenyl diselenide (0.22 mmol), *t*-BuOK (0.4 mmol), and DMF (2 ml, 0.1 M) was added in a 5 mL glass tube, which was stirred at room temperature for 0.5 h. When the reaction was completed, the next steps were the same as those described above to obtain the desired product SYG-180-2-2 (Figure 1). The full name of SYG-180-2-2 is *N*-(3-(phenylselanyl)-1-pivaloyl-1*H*-indol-7-yl) benzamide.

Next, 7-amido indoles 2 (0.2 mmol), Pd (TFA)_2_ (5 mol%), AgOAc (0.6 mmol), and PivOH (1.2 mmol) were added into a 12 mL screw capped tube with 2 mL of benzene at room temperature. The reaction mixture was allowed to warm up to 110°C and stirred for 4 h. When the reaction was completed, the next steps were the same as those described above to obtain the desired product SYG-180-7 (Supplementary Figure 1). The full name of SYG-180-7 is *N*-(2-phenyl-1-pivaloyl-1*H*-indol-7-yl) benzamide.

### Bacterial Strains, Cells, and Growth Conditions

Bacterial strains used in this study are described at Table 1. Methicillin-resistant *S. aureus* strains JP5023 and JP4856 were isolated from patients with different infection sites at the First Affiliated Hospital of Wenzhou Medical University. On the basis of their ability to form potent biofilm, we used them to carry out biofilm research. We used Trypticase soy broth (TSB, BD Biosciences, Franklin Lakes, NJ, United States) medium without antibiotics to culture all strains at 37°C with shaking at 220 rpm.

**TABLE 1:** Bacterial strains used in this study.

Strain	SYG-180-2-2 MIC (μg/ml)	Source	Ward	Antibiotic resistance/susceptibility profiles
JP5023	>128	Blood	Emergency rescue	PG^1^ (R); OX^2^ (R); EM^3^ (R); CC^4^ (R); CIP^5^ (I)
JP4856	>128	Pus	Otolaryngology department	PG (R); OX (R); EM (R); CC (R); CIP (S)

*1: Penicillin G; 2: Oxacillin; 3: Erythromycin; 4: Clindamycin; 5: Ciprofloxacin.*

Human normal alveolar epithelial cells BEAS-2B were a gift from the Clinical Transformation Center, Shanghai Pulmonary Hospital, Tongji University School of Medicine and cultured in Dulbecco’s Modified Eagle’s Medium [DMEM, Thermo Fisher Biochemical Products (Beijing) Co., Ltd.].

### Determination of Minimum Inhibitory Concentration

SYG-180-2-2 was diluted with dimethyl sulfoxide (DMSO, Biosharp, Beijing, China) to the concentration of 20 mg/ml. The MIC values of SYG-180-2-2 against JP5023 and JP4856 were determined by the microtiter broth dilution method (van Hal et al., 2011). The colonies were cultured for 16–18 h and directly extracted to prepare a 0.5 MacFarland turbidity standard bacterial suspension, and then diluted with cation-adjusted Mueller-Hinton broth (CAMHB) 1:100. A total of 100 μl of medium containing SYG-180-2-2 (1–128 μg/ml) and 100 μl of suspension were added into a 96-well microfilter plate. In the experiment, we used DMSO as a control. After that the plate was incubated for 16–18 h at 37°C. All assays were performed in triplicate. The minimum concentration at which no bacterial growth was observed by the naked eye was defined as the MIC.

### Growth Inhibition Assay

Methicillin-resistant *S. aureus* strains were grown in TSB for 4–6 h and made into a bacterial suspension with a turbidity of 0.5 MacFarland standard. Then we performed 1:100 dilution into TSB medium containing SYG-180-2-2, so that the final concentrations of the medium were 4 and 8 μg/ml. No drug was added as a positive control, TSB was the negative control. An equivalent volume of DMSO to the 4 and 8 μg/ml SYG-180-2-2 samples was used as a control in the experiment in order to exclude the influence of solvent on bacterial growth. A 200 μl mixed liquor was added to a sterile bioscreen honeycomb plate. We used an automatic microbial growth curve analyzer (OY Growth Curves, Finland) to measure OD_600_ every 1 h for 24 h and obtain a growth curve according to the measured values. The test was performed in triplicate.

### Biofilm Formation Assessment

Overnight-cultured MRSA strains JP5023 and JP4856 were diluted 1:100 in different drug concentrations (0–32 μg/ml) with TSB containing 0.5% glucose (TSBG), and each concentration was added to three parallel wells in 96-well microplates. After incubation for 24 h, the wells were washed carefully three times with 200 μl of phosphate-buffered saline [PBS, Sangon Biotech (Shanghai) Co., Ltd.]. Removing unattached bacteria, biofilms were fixed with 200 μl of 99% methanol for 15 min and stained with 200 μl of 1% crystal violet for 8 min (Chaieb et al., 2011). The excess dye was gently washed off the wells with running water until the water was colorless. The absorbance was measured at 600 nm after adding 30% acetic acid.

### Biofilms Observed by Laser Scanning Confocal Microscopy

Strains were incubated by TSBG in 20 mm glass-bottomed cell culture dishes (NEST, Wuxi, China). After 24 h, we washed the dishes twice with PBS to remove floating cells and then added SYTO-9 (0.02%, Thermo Fisher Scientific, Waltham, MA, United States) and PI (0.067%, Thermo Fisher Scientific, Waltham, MA, United States) to stain biofilms for 30 min in the dark. After staining, samples were scanned by CLSM (TCS SP5; Leica, Wetzlar, Germany) using a 63 × oil immersion objective lens directly.

### Bacterial Biofilm Metabolism Activity

3-(4,5-Dimethylthiazol-2-yl)-2,5-diphenyltetrazolium bromide, also called MTT, is reduced to the water-insoluble blue-purple formazan by amber dehydrogenase in the mitochondria of living cells. Formazan is dissolved by DMSO, and then its absorbance can indirectly reflect the number of living bacteria. We used MTT to detect the biofilm metabolism activity. In brief, overnight-cultured MRSA strains JP5023 and JP4856 were diluted 1:100 with TSBG containing 4 μg/ml of SYG-180-2-2 in 96-well plates, wells without SYG-180-2-2 were control. Each condition was tested in three replicate wells. Plates were incubated at 37°C for 6, 12, 24, and 48 h, respectively. We removed the supernatant and washed the wells twice with PBS. Then, 100 μl of TSBG containing 0.25 mg/ml MTT (Beijing Solarbio Science and Technology Co., Ltd.) was added into each well and incubated at 37°C for 0.5 h in dark. Subsequently, the supernatant was discarded and 100 μl of DMSO was added to wells to dissolve biofilms, and then the optical density of the wells was measured at OD_490_.

### Cell Adhesion Assay

The experimental method was slightly modified according to the previously described method (Wang et al., 2021). Briefly speaking, after MRSA strains were cultured overnight in TSB containing 2% glucose, 100 μl of the overnight culture was added to 96-well plates. Subsequently, the equal volume of TSB including SYG-180-2-2 and SYG-180-7 was added, respectively, to realize the desired final concentration of 4 μg/ml. The plates were incubated at 37°C for 4 h. After this, the plate was washed with PBS to discard the floating cells and the absorbance was measured at 600 nm. SYG-180-7 was severed as a control compound to exclude the possibility that the hydrophobicity of the compound itself inhibits the interaction between MRSA and the sold surface.

### Polysaccharide Intercellular Adhesin and Extracellular DNA Detection

For polysaccharide intercellular adhesin (PIA) detection, we diluted the overnight culture 1:100 in 3 ml TSBG containing a concentration of 4 μg/ml SYG-180-2-2 into a six-well plate at 37°C for 24 h, wells without SYG-180-2-2 served as the control. Planktonic cells were removed and washed with PBS, then biofilms were resuspended with 500 μl of 0.5 M EDTA [PH 8.0, Sangon Biotech (Shanghai) Co., Ltd.] using a scraper. Cells were incubated at 100°C for 5 min and centrifuged at 12,000 rpm for 2 min. Then, 40 μl of supernatant was added to 20 μl of proteinase K (20 mg/ml) at 37°C for 2 h. A total of 10 μl of the treated PIA sample was spotted onto the polyvinylidene fluoride (PVDF) membrane which was activated by methanol. The membrane was kept moist and smooth during the spotting process. After drying, the membrane was blocked with 3.5% bovine serum albumin (BSA) (Biosharp, Beijing, China) in PBS with 0.1% Tween 20 (PBST) [Sangon Biotech (Shanghai) Co., Ltd.] at 4°C overnight, and incubated at 37°C with Wheat Germ Agglutinin-HRP (WGA-HRP) conjugate for 1 h at a Universal Antibody Diluent (New Cell and Molecular Biotech Co., Ltd.) of 1:5,000. The membrane was washed thoroughly three times with PBST and detected using enhanced chemiluminescence (ECL) (Affinity Bio, San Francisco, CA, United States).

For extracellular DNA (eDNA) detection, MRSA strains were cultured in six-well plates as described above. After incubation at 37°C for 24 h, the eDNA was extracted as previously described (Rice et al., 2007). The amount of eDNA was measured using a UV Nanodrop 2000 (ThermoFisher Scientific Ltd.). The experiment was repeated three times.

### Isolation of RNA and Quantitative RT-PCR

We followed the manufacturer’s instructions [(Spin Column Bacteria Total RNA Purification Kit and Sangon Biotech (Shanghai) Co., Ltd.] for RNA extraction. Briefly, MRSA strains were cultured in TSB with and without SYG-180-2-2 at 37°C for 16 h. The bacterial mass was collected by centrifugation and suspended in lysozyme (20 mg/ml) and lysostaphin (1 mg/ml) at 37°C for 1 h. Then total RNA was extracted and cDNA was synthesized using a Primescript™ RT reagent Kit with gDNA Eraser (Takara, Tokyo, Japan).

Quantitative real-time PCR (qPCR) was performed using the Fast Start DNA Master SYBR Green II Mixture (Takara, Tokyo, Japan) and QuantStudio^®^ 5 Applied Biosystems (ABI) Fluorescence quantitative PCR instrument (Thermo Fisher Scientific). The reaction used the DNA sequence of gyrB as an internal reference and was performed in a 20 μl reaction volume per well. Table 2 shows the primer pairs used for RT-PCR. The cycling conditions were 95°C for 30 s, followed by 40 cycles, with 1 cycle consisting of 95°C for 5 s and 60°C for 34 s. The cycle threshold (Ct) measurements were calculated by the QuantStudio™ Design and Analysis SE software version 1.6.0. First, the relative expression levels of biofilm-related genes treated with and without SYG-180-2-2 were normalized to the *gyrB* reference gene to obtain ΔCt_1_ and ΔCt_2_, respectively. ΔΔCt was acquired by subtracting ΔCt_2_ from ΔCt_1_. Then we used the relative quantification method (2^–ΔΔCt^) to analyze the transcription level of the target gene in the sample with SYG-180-2-2. Three replicates were performed for each condition.

**TABLE 2:** Primes used in this study.

Primer	Sequence (5′–3′)
*gyrB*-RT-F	ACATTACAGCAGCGTATTAG
*gyrB*-RT-R	CTCATAGTGATAGGAGTCTTCT
*icaA*-RT-F	GTTGGTATCCGACAGTATA
*icaA*-RT-R	CACCTTTCTTACGTTTTAATG
*icaR*-RT-F	GGATGCTTTCAAATACCAACT
*icaR*-RT-R	TTATCTAATACGCCTGAGGAAT
*codY*-RT-F	GACAATGTATTAACAGTATTCC
*codY*-RT-R	TAGCAGCATATTCACCTA
*fnbB*-RT-F	GCGAAGTTTCTACTTTTG
*fnbB*-RT-R	CAACCATCACAATCAACA
*eno*-RT-F	CTCCAATTGCATTCCAAG
*eno*-RT-R	GCATCTTCAGTACCTTCA
*fib*-RT-F	GTGCTTTACGGTGTGTTG
*fib*-RT-R	CTGCTATTAGTTTAACGGTATCAA
*ebpS*-RT-F	GTGTGATGATTCGACTTG
*ebpS*-RT-R	CAGGATACAATAGAGAATACG
*saeR*-RT-F	GTCGTAACCATTAACTTCTG
*saeR*-RT-R	ATCGTGGATGATGAACAA
*psm*α-RT-F	ATGGAATTCGTAGCAAAATTATTC
*psm*α-RT-R	TAGTTGTTACCTAAAAATTTACC
*psm*β-RT-F	CCTAGTAAACCCACACCG
*psm*β-RT-R	GCTGCACAACAACATGATA
*agrA*-RT-F	GCAGTAATTCAGTGTATGTTCA
*agrA*-RT-R	TATGGCGATTGACGACAA

### Assessment of SYG-180-2-2 Cytotoxicity

The Cell Counting Kit-8 (CCK-8) solution was used to evaluate the proliferation and cytotoxicity of SYG-180-2-2 to BEAS-2B (Yu et al., 2017). WST-8 is reduced by cellular dehydrogenase to an orange formazan product which can dissolve in culture medium. The amount of formazan product is directly proportional to the number of living cells. In short, the cells were seeded in a 96-well plate at different cells/well for 12 h. Then we discarded the supernatant and added a final concentration of 4 μg/ml of SYG-180-2-2 DMEM containing 10% fetal bovine serum (FBS, Sigma-Aldrich, St. Louis, MO, United States) and 1% penicillin/streptomycin solution (sterile), wells without SYG-180-2-2 were used as a positive control. After 24 h, the supernatant was removed and wells were washed twice with PBS. Then 100 μl of DMEM and 10 μl of cck8 were added to wells at 37°C for 1–2 h. Finally, 450 nm absorbance was measured. Three independent experiments were carried out.

### Statistical Analysis

Statistical analysis was performed using GraphPad Prism (version 8.0). Multiple *t*-tests were used for the growth curve. Biofilm formation assessment was analyzed using one-way analysis of variance (ANOVA) followed by Tukey’s multiple-comparison test. Unpaired two-tailed *t*-tests were used for the other experiments. *P* values < 0.05 were considered statistically significant. *P < 0.05, ^**^P < 0.01, ^***^P < 0.001, and ^****^P < 0.0001. All figures were presented as mean ± standard deviation.

## Results

### Characterization of Products SYG-180-2-2 and SYG-180-7

**FIGURE 2:**
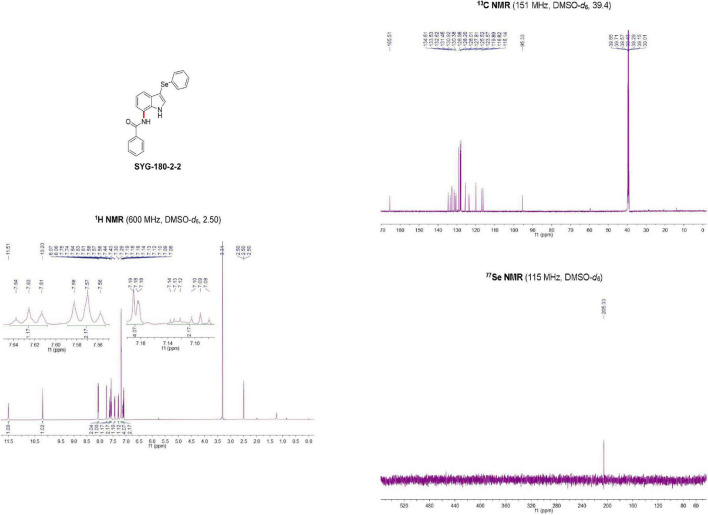
Nuclear magnetic resonance (NMR) identification of SYG-180-2-2.

**FIGURE 3:**
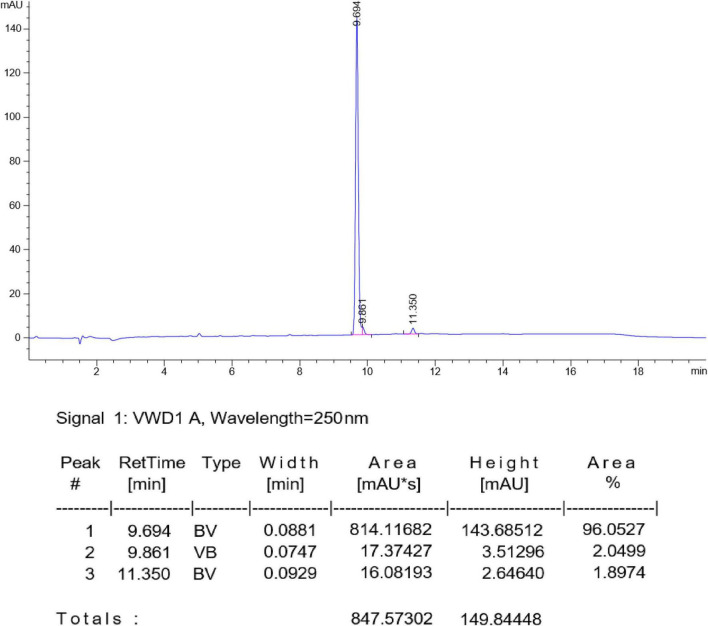
High-resolution mass spectrometry (HRMS) identification of SYG-180-2-2.

SYG-180-2-2 was a white solid (96.0527% purity) after purification by chromatography (elution: 35% EtOAc in petroleum ether) with a melting point of 230–231°C. SYG-180-2-2 was characterized by nuclear magnetic resonance (NMR, Figure 2) spectroscopy and high-resolution mass spectrometry (HRMS, Figure 3), obtaining the following results: ^1^H NMR (600 MHz, DMSO-*d*_6_) δ 11.51 (brs, 1H), 10.20 (brs, 1H), 8.07 (d, *J* = 7.3 Hz, 2H), 7.74 (d, *J* = 2.6 Hz, 1H), 7.63 (t, *J* = 7.3 Hz, 1H), 7.57 (t, *J* = 7.5 Hz, 2H), 7.43 (d, *J* = 7.5 Hz, 1H), 7.29 (d, *J* = 7.9 Hz, 1H), 7.20–7.18 (m, 4H), 7.14–7.08 (m, 2H) ppm;^13^C NMR (151 MHz, DMSO-*d*_6_) δ 165.51, 134.61, 133.53, 132.62, 131.45, 130.92, 130.38, 128.98, 128.20, 128.01, 127.81, 125.52, 123.57, 119.89, 116.82, 116.14, and 95.33 ppm; ^77^Se NMR (115 MHz, DMSO-*d*_6_) δ 205.33 ppm; HRMS: calc. for C_21_H_17_N_2_OSe^+^ [M + H]^+^: 393.05061, found: 393.05005.

SYG-180-7 was a white solid (100% purity) after purification by chromatography (elution: 15% EtOAc in petroleum ether) with a melting point of 125–126°C. SYG-180-7 was characterized by nuclear magnetic resonance (NMR, Supplementary Figure 2) spectroscopy and high-resolution mass spectrometry (HRMS, Supplementary Figure 3), obtaining the following results: ^1^H NMR (600 MHz, CDCl_3_) δ 8.27 (brs, 1H), 8.04 (d, *J* = 7.3 Hz, 2H), 7.97 (d, *J* = 7.8 Hz, 1H), 7.60–7.53 (m, 5H), 7.48 (d, *J* = 7.8 Hz, 1H), 7.45 (t, *J* = 7.6 Hz, 2H), 7.39 (t, *J* = 7.4 Hz, 1H), 7.29–7.26 (m, 1H), 6.77 (s, 1H), 0.75 (s, 9H) ppm. ^13^C NMR (151 MHz, CDCl_3_) δ 193.1, 164.8, 139.9, 134.1, 133.0, 131.9, 130.5, 129.1, 128.8, 128.7, 128.6, 128.1, 127.1, 123.0, 122.1, 119.6, 117.9, 106.2, 46.6, and 27.6 ppm. HRMS (ESI) m/z: [M + H]^+^ calc. for C_26_H_25_N_2_O_2_: 397.1916; found, 397.1914.

### Influence of Subinhibitory Concentrations of SYG-180-2-2 on the Growth of Methicillin-Resistant *Staphylococcus aureus* Strains

**FIGURE 4:**
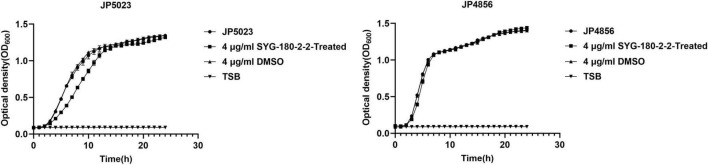
Growth curves of methicillin-resistant *Staphylococcus aureus* (MRSA) strains treated with SYG-180-2-2. Strains JP5023 and JP4856 were cultured with 4 μg/ml of or without SYG-180-2-2. Trypticase soy broth (TSB) was used as a blank control. Dimethyl sulfoxide (DMSO) was used as a control in order to exclude the influence of solvent on bacterial growth.

The minimum inhibitory concentration (MIC) values of SYG-180-2-2 against MRSA JP5023 and JP4856 were >128 μg/ml. According to the growth curve we drew, the amount of MRSA strain JP4856 in the late logarithmic growth period was consistent at the subinhibitory concentration of 4 μg/ml. But, at 4 μg/ml, MRSA strain JP5023 grew more slowly than bacteria in the control wells at 4–11 h, and the growth was consistent in the late logarithmic phase (Figure 4). The high concentration of SYG-180-2-2 (8 μg/ml) inhibited the growth of MRSA JP5023 (Supplementary Figure 4).

### SYG-180-2-2 Inhibits Methicillin-Resistant *Staphylococcus aureus* Biofilm Formation

**FIGURE 5:**
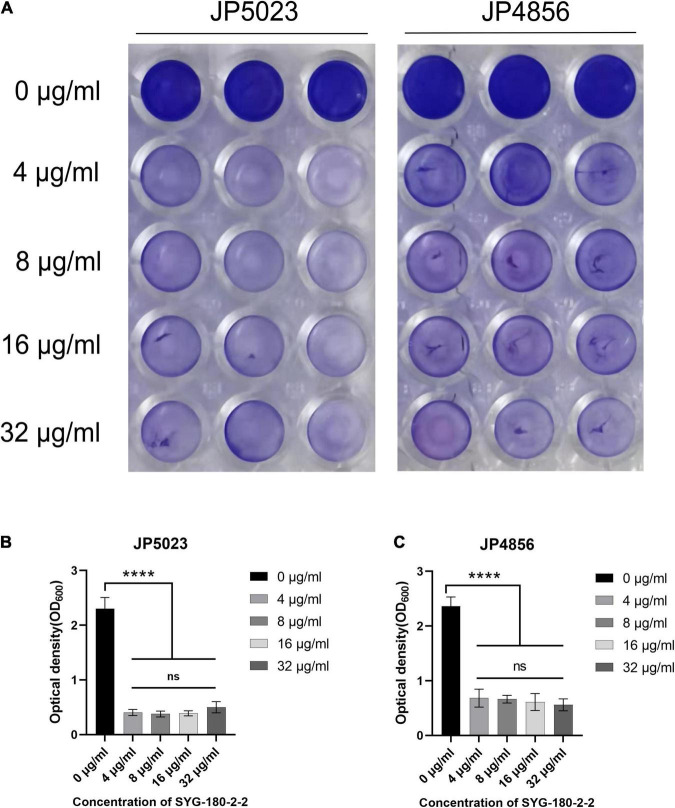
The effect of SYG-180-2-2 on the formation of biofilm. **(A)** Biofilm formation in a 96-well plate. At OD_600_, there was a significant difference in the biofilm formation of JP5023 **(B)** and JP4856 **(C)** cultured with or without SYG-180-2-2. ****P < 0.0001.

**FIGURE 6:**
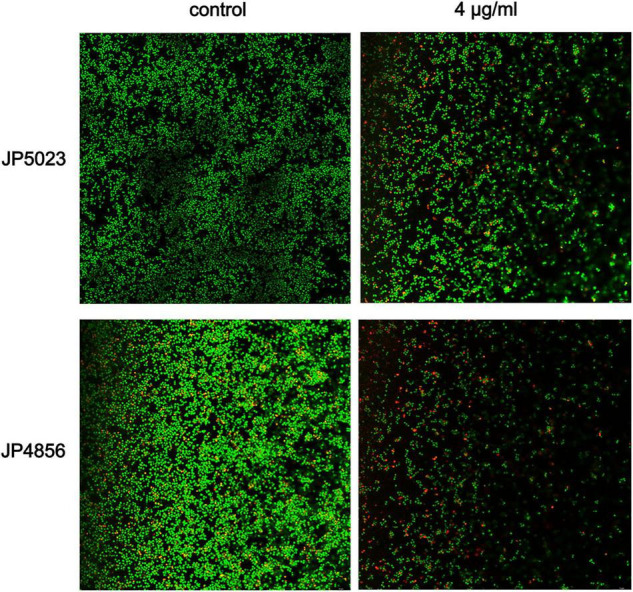
Biofilm formation was observed by CLSM. JP5023 and JP4856 treated with SYG-180-2-2 (4 μg/ml).

Bacterial biofilms are difficult to eradicate and resistant to antibacterial drugs (Tan et al., 2012). We used semi-quantitative biofilm to detect the effect of subinhibitory concentrations of SYG-180-2-2 on MRSA biofilm. Treatment with SYG-180-2-2 at a concentration of 4 μg/ml decreased the JP5023 and JP4856 biofilm by 82.9 ± 2.3 and 71.9 ± 6.8%, respectively, when compared with the untreated group (Figure 5). Similarly, treatment with SYG-180-2-2 at concentrations of 8, 16, and 32 μg/ml had a significant reduction effect on biofilms. These results showed that sub-MICs of SYG-180-2-2 (4, 8, 16, and 32 μg/ml) were not affected by dose. We observed in the bacterial biofilm treated with SYG-180-2-2 by CLSM at 4 μg/ml that the density of the biofilm was lower and sparser (Figure 6), compared with the untreated group.

### SYG-180-2-2 Reduces the Metabolic Activity of Methicillin-Resistant *Staphylococcus aureus* Biofilm

**FIGURE 7:**
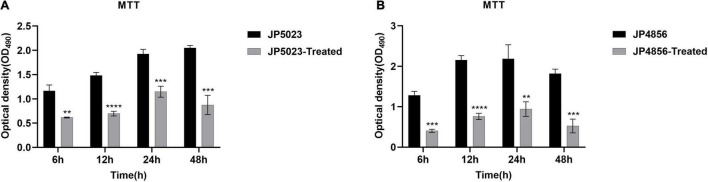
The effect of subinhibitory concentration of SYG-180-2-2 on the metabolic activity of MRSA strains JP5023 **(A)** and JP4856 **(B)** biofilm. **P < 0.01, ***P < 0.001, and ****P < 0.0001.

Under the action of a subinhibitory concentration of SYG-180-2-2 (4 μg/ml), the bacterial metabolic activity of the strains was measured in four time points by MTT staining. Reduction of the metabolic activity of JP5023 strain in the presence of SYG-180-2-2 at 4 μg/ml after 6, 12, 24, and 48 h were 47.2 ± 0.6, 53.3 ± 3.6, 40.3 ± 6.6, and 57.6 ± 9.9%, respectively, when compared to the untreated groups. At the same time, reduction of the metabolic activity of JP4856 strain after 6, 12, 24, and 48 h of SYG-180-2-2 treatment with 4 μg/ml were 71.0 ± 2.7, 64.8 ± 3.5, 57.0 ± 8.5, and 71.2 ± 7.3%, respectively, compared to the control (Figure 7).

### SYG-180-2-2 Affects the Adhesion of Methicillin-Resistant *Staphylococcus aureus*

**FIGURE 8:**
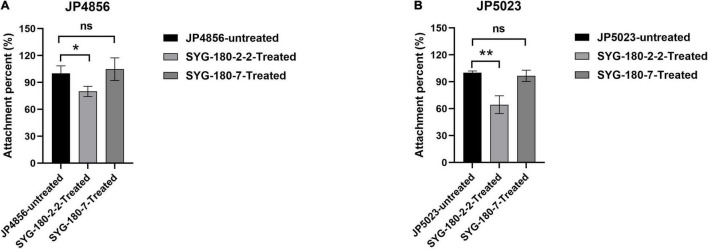
Attachment percent of JP5023 **(A)** and JP4856 **(B)** explored by attachment assay (the control was set to 100%). *P < 0.05 and **P < 0.01.

We observed the effect of a subinhibitory concentration of SYG-180-2-2 (4 μg/ml) on the initial adhesion stage of MRSA biofilm by an attachment assay. The results showed that SYG-180-2-2 significantly suppressed the adhesion of JP5023 and JP4856 to the solid surface at 4 μg/ml by 37.6 ± 9.3 and 21.4 ± 5.1%, respectively (Figure 8). Meanwhile, SYG-180-7 showed no significant difference in cell adhesion, when compared with the untreated strains (Figure 8).

### The Effect of SYG-180-2-2 on the Production of Polysaccharide Intercellular Adhesin and Extracellular DNA in Methicillin-Resistant *Staphylococcus aureus*

**FIGURE 9:**
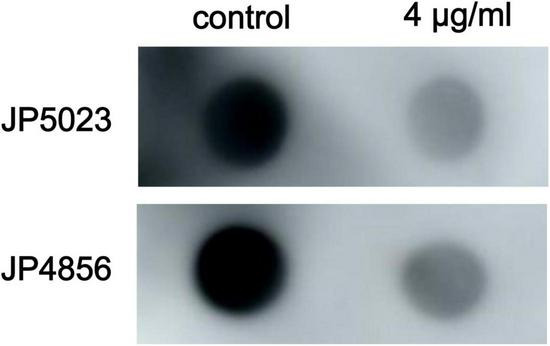
Effect of the subinhibitory concentration of SYG-180-2-2 on MRSA PIA production.

In order to study the effect of SYG-180-2-2 on the biofilm matrix of MRSA, the release of PIA and eDNA was detected. Compared with the untreated group, the production of PIA with SYG-180-2-2-treated strains was decreased significantly (Figure 9), however, there was no significant difference in eDNA (Supplementary Figure 5).

### Effect of SYG-180-2-2 on the Expression of Biofilm-Related Genes

**FIGURE 10:**
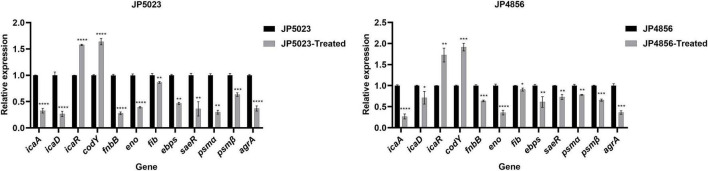
The effect of MRSA strains with SYG-180-2-2 treatment on biofilm-related gene expression. *P < 0.05, **P < 0.01, ***P < 0.001, and ****P < 0.0001.

The transcript levels of biofilm-related genes treated with the concentration of 4 μg/ml of SYG-180-2-2 were determined using RT-PCR to clarify the effect of SYG-180-2-2 on the formation of biofilm. In general, the results showed that in JP5023 and JP4856, except for the expression of *icaR* and *codY* genes which was upregulated, the expression of *icaA*, *icaD*, *icaR*, *fnbB*, *eno*, *fib*, *ebps*, *saeR*, *psm*α, *psm*β, and *agrA* genes was downregulated to varying degrees with the treatment of SYG-180-2-2 (Figure 10). These results were consistent with the adhesion of bacteria and the detection of PIA.

### Subinhibitory Concentration of SYG-180-2-2 Is Non-toxic to Human Alveolar Epithelial Cells

**FIGURE 11:**
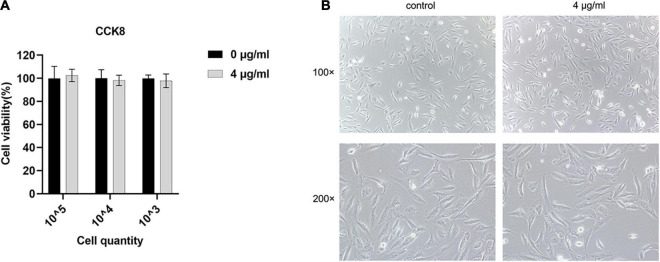
The effect of SYG-180-2-2 on BEAS-2B. **(A)** The activity of different amounts of BEAS-2B with or without SYG-180-2-2 treatment. **(B)** Microscopic cell morphology.

In order to study the effect of SYG-180-2-2 on human cytotoxicity, we used BEAS-2B in our experiments to evaluate the cytotoxicity of SYG-180-2-2 with the CCK-8 assay. There was no effect on the cytotoxicity when SYG-180-2-2 was used (Figure 11A). When the cells were seeded at 3,000 cells/well, the cell morphology was not abnormal under the microscope (Figure 11B). Obviously, SYG-180-2-2 is not cytotoxic at a subinhibitory concentration.

## Discussion

When MRSA strains acquire resistance to antibiotics and form robust biofilm, this leads to higher mortality, especially when they infect patients in the intensive care unit (ICU; Turner et al., 2019). Fortunately, we synthesized a new small-molecule compound SYG-180-2-2 that possessed significant inhibitory activity against the biofilm of MRSA ranging from different types.

In recent years, there have been many reports on the effect of antibacterial drugs with subinhibitory concentrations on biofilms (Goneau et al., 2015). SYG-180-2-2 has a higher MIC, however, at low concentrations, proving it has remarkable anti-biofilm activity. At 4 μg/ml, the amount of JP5023 slowed down in the logarithmic phase; the possible reason was that the bacteria incurred the cost of adaptability for growth. A higher concentration of SYG-180-2-2 suppressed the growth of JP5023, while at 4 μg/ml, it had no effect on the later growth of the bacteria. Hence, the growth curve proved that the biofilm inhibitory effects of SYG-180-2-2 (4 μg/ml) were not due to its bactericidal efficacy. We speculate that the higher the sub-MICs (8, 16, and 32 μg/ml) of SYG-180-2-2, the more likely bactericidal efficacy is to attenuate the biofilms. In biofilm formation assessment, there were no significant differences among the subinhibitory concentrations (4, 8, 16, and 32 μg/ml). Therefore, through the growth curve and biofilm formation assay, we focused on the lower concentration of 4 μg/ml in the experiment in order to exclude that SYG-180-2-2 inhibits MRSA biofilm formation by preventing cell proliferation. In addition to semi-quantitative biofilm experiments, CLSM further confirmed that the subinhibitory concentration of SYG-180-2-2 could reduce biofilm formation significantly in clinical isolates of MRSA. A metabolic assay is a brilliant method to quantify the viability of bacteria in biofilms. The number of living bacteria in the biofilm and the metabolic activity of individual bacteria determine the quantity of metabolites produced by the biofilm (Kot et al., 2019). As CV-stained biomass contains dead bound bacteria instead of live bacteria, we used MTT to detect the amount of live bacteria in the biofilm at the same time, which proved that SYG-180-2-2 has anti-biofilm activity.

Primary attachment is the first step for bacteria to bind to the host surface. In our experiment, SYG-180-7, which has a similar structure to SYG-180-2-2, was used as the control, indicating that SYG-180-2-2 inhibits the binding of MRSA to the host surface due to its anti-adhesion rather than hydrophobicity. The decreased expression of *fnbB*, *fib*, *ebpS*, and *eno* genes involved in adhesion further proved that SYG-180-2-2 could prevent the initiation of host tissue colonization. PIA and eDNA are essential for biofilm formation (Lopez et al., 2010). It was reported that PIA-dependent biofilm often appears in methicillin-sensitive *S. aureus* (MSSA), while PIA-independent biofilm is common in MRSA (Nguyen et al., 2020). In contrast, the strains we used in the experiment produced large amounts of PIA. PIA-dependent biofilm formation results in a stronger and steadier biofilm than those whose biofilm is PIA-independent (Rohde et al., 2007; Dice et al., 2009). In our study, we found that SYG-180-2-2 could reduce the PIA production to inhibit biofilm formation, while the production of eDNA was not significantly decreased. These results indicate that the formation of *S. aureus* biofilm may be prevented by affecting the PIA production rather than eDNA. Both *ica*-negative and *ica*-positive MRSA can produce biofilm, and the extracellular matrix of *ica*-positive MRSA is mainly composed of PIA, while those of *ica*-negative MRSA is mostly formed of eDNA (Chopra et al., 2015). It is well known that the *ica* operon affects the formation of PIA (Nguyen et al., 2020), which is confirmed by the decrease in the expression of *icaA* and *icaD* according to the RT-qPCR method. Meanwhile, the expression of *icaR* was upregulated. PIA is the main influence of low CodY activity bacteria on biofilm formation, and most recent works showed that *codY* regulated the PIA-dependent biofilm (Majerczyk et al., 2008; Atwood et al., 2015; Waters et al., 2016; Schilcher and Horswill, 2020). In our study, we guess the upregulation of *codY* prevented PIA production. Moreover, *saeR* is not only a key regulator of virulence gene expression (Nagel et al., 2018), but also affects the maturation process of biofilm (Mashruwala et al., 2017). The downregulation of its expression indicated that SYG-180-2-2 may also have an effect on biofilm maturation and virulence. *Psms* including *psm*α and *psm*β are considered to disperse biofilm resulting in persistent infection (Periasamy et al., 2012). Furthermore, the *psms* gene is positively regulated by the *agrA* gene (George et al., 2019). Our results were consistent with the above, when the expression of *agrA* decreased, as did the expression of *psms*. Taken together, SYG-180-2-2 inhibits biofilm formation by preventing the adhesion of bacteria and the production of PIA.

More importantly, the subinhibitory concentration of SYG-180-2-2 (4 μg/ml) is not only non-toxic to human cells but can also inhibit the formation of biofilm. We concluded that SYG-180-2-2 had the potential to become a new type of antimicrobial drug used in clinical practice.

## Data Availability Statement

The original contributions presented in the study are included in the article/Supplementary Material, further inquiries can be directed to the corresponding authors.

## Author Contributions

LR, YS, and YX designed the work and analyzed and interpreted the data for the work. LR and JY drafted the work and revised it critically for important intellectual content. BW, HZ, XWa, XWu, YG, and ZS participated in the experimental design and data analysis. FY and LZ provided approval for publication of the content and agreed to be accountable for all aspects of the work in ensuring that questions related to the accuracy or integrity of any part of the work are appropriately investigated and resolved. All authors read and approved the final manuscript.

## Conflict of Interest

The authors declare that the research was conducted in the absence of any commercial or financial relationships that could be construed as a potential conflict of interest.

## Publisher’s Note

All claims expressed in this article are solely those of the authors and do not necessarily represent those of their affiliated organizations, or those of the publisher, the editors and the reviewers. Any product that may be evaluated in this article, or claim that may be made by its manufacturer, is not guaranteed or endorsed by the publisher.

## Supplementary Material

Supplementary Figure 1 Synthetic process of SYG-180-7. 1: *N*-pivaloyl indoles. 2: *N*-(1-pivaloyl-1*H*-indol-7-yl) benzamide. SYG-180-7: desired product.

Supplementary Figure 2 Nuclear magnetic resonance (NMR) identification of SYG-180-7.

Supplementary Figure 3 High-resolution mass spectrometry (HRMS) identification of SYG-180-7.

Supplementary Figure 4 Growth curves of MRSA strains cultured with SYG-180-2-2 (8 μg/ml).

Supplementary Figure 5 Effect of the subinhibitory concentration of SYG-180-2-2 on MRSA eDNA production.
