# Supplementary figures and images for: Small-Molecule Compound SYG-180-2-2 to Effectively Prevent the Biofilm Formation of Methicillin-Resistant Staphylococcus aureus
Source: Front Microbiol. 2022 Jan 7;12:770657. doi: 10.3389/fmicb.2021.770657 (PMC8777106; doi:10.3389/fmicb.2021.770657)

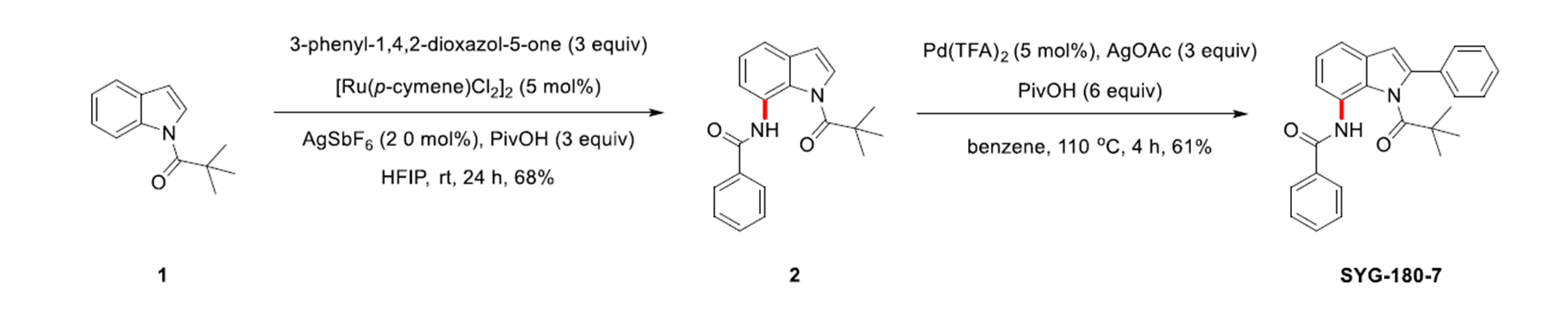

Supplement: Supplementary Figure 1 — Synthetic process of SYG-180-7. 1: N-pivaloyl indoles. 2: N-(1-pivaloyl-1H-indol-7-yl) benzamide. SYG-180-7: desired product. [file Image_1.TIF]

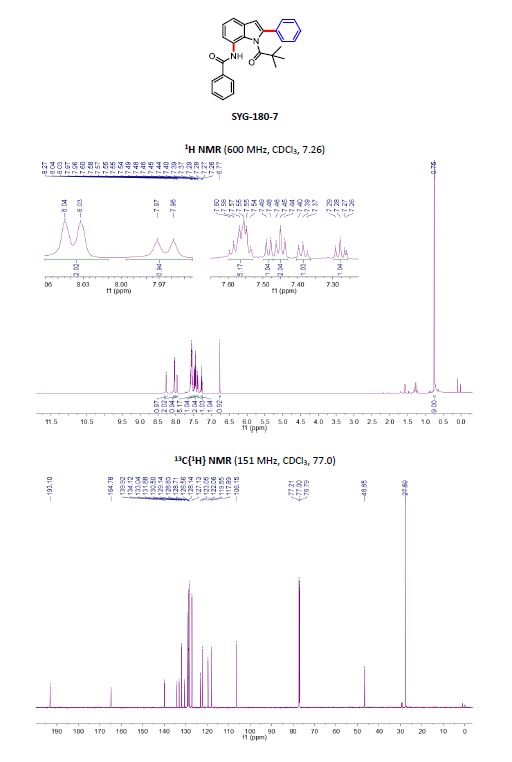

Supplement: Supplementary Figure 2 — Nuclear magnetic resonance (NMR) identification of SYG-180-7. [file Image_2.TIF]

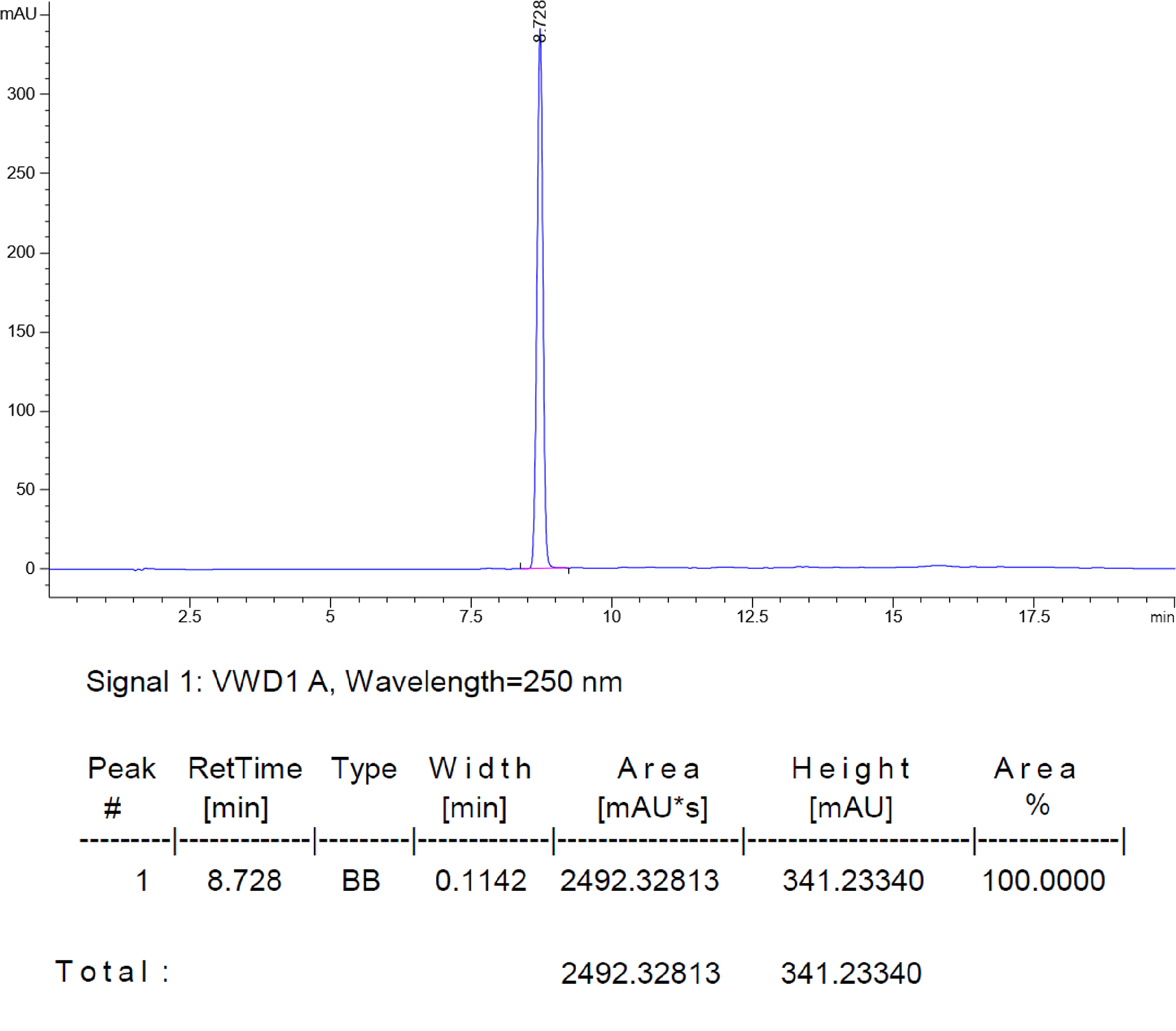

Supplement: Supplementary Figure 3 — High-resolution mass spectrometry (HRMS) identification of SYG-180-7. [file Image_3.TIF]

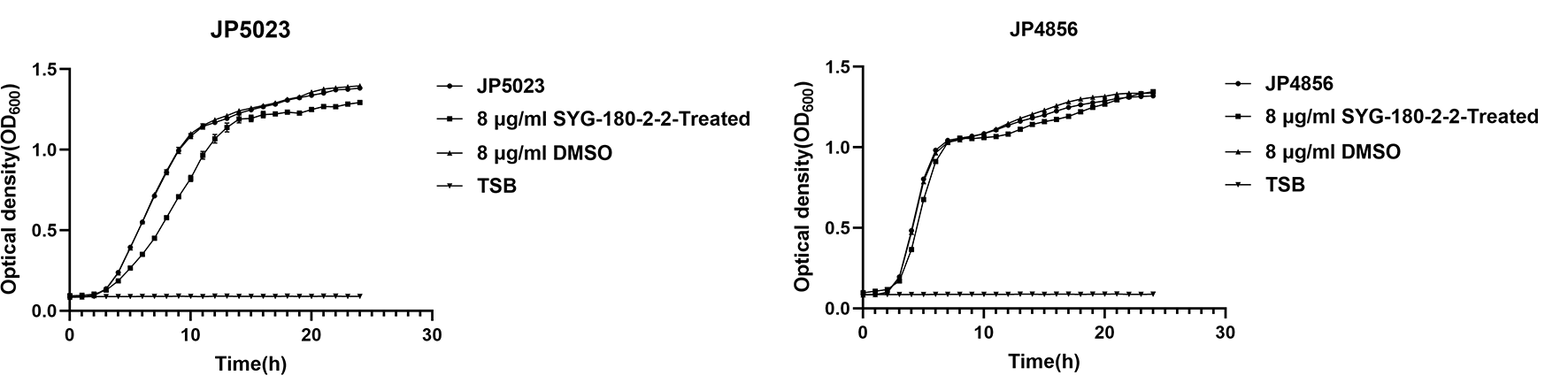

Supplement: Supplementary Figure 4 — Growth curves of MRSA strains cultured with SYG-180-2-2 (8 μg/ml). [file Image_4.TIF]

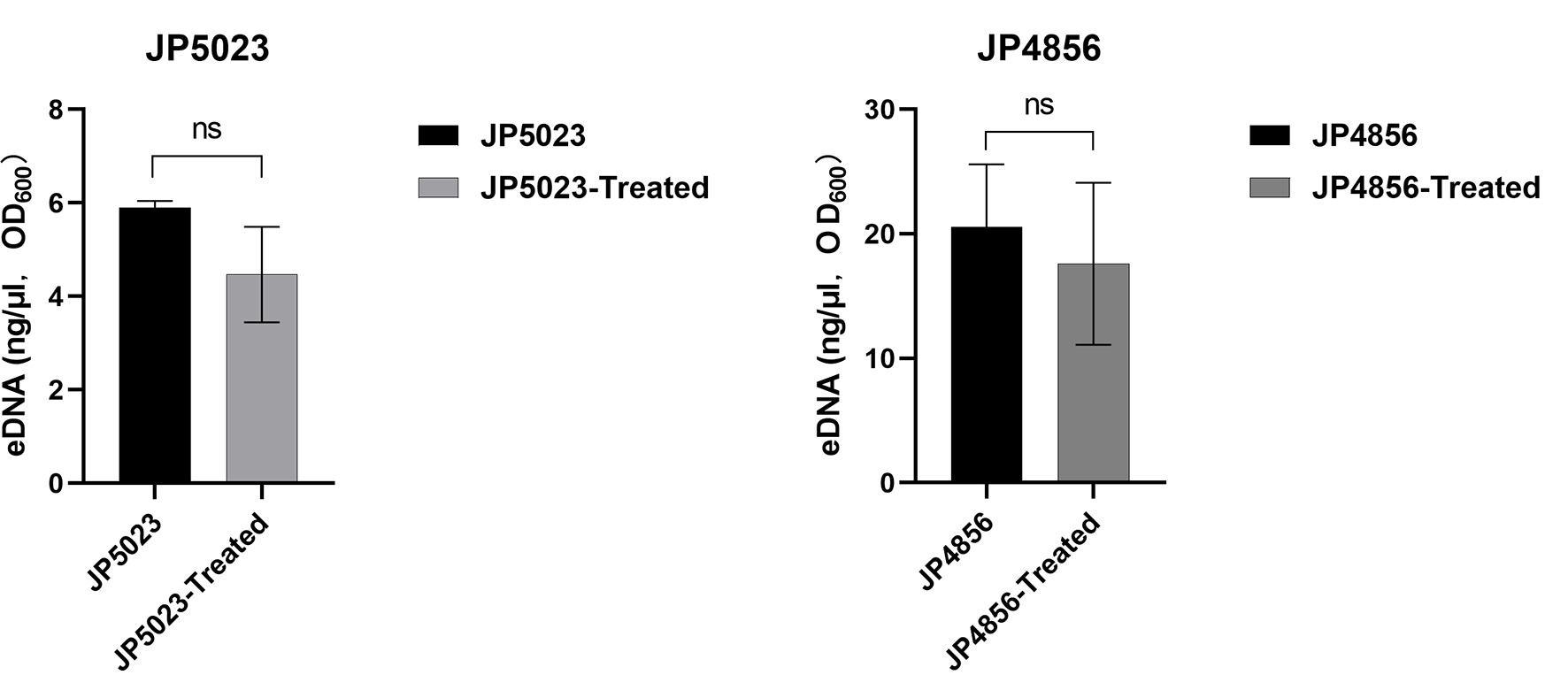

Supplement: Supplementary Figure 5 — Effect of the subinhibitory concentration of SYG-180-2-2 on MRSA eDNA production. [file Image_5.TIF]
